# Infectious Thrombophlebitis of the Left Internal Jugular Vein With Tissierella praeacuta Isolation in a 71-Year-Old Female: A Case Report

**DOI:** 10.7759/cureus.67478

**Published:** 2024-08-22

**Authors:** Shawn Howell, Morgan Gurel-Headley, Leonard Ferdman, Christian Ratzlaff

**Affiliations:** 1 Internal Medicine, University of Arkansas for Medical Sciences, Fayetteville, USA

**Keywords:** lemierre's syndrome, tissierella praeacuta, thrombus, infectious thrombophlebitis, internal jugular thrombus

## Abstract

*Tissierella praeacuta*, a rare pathogen, was found in a 71-year-old female's internal jugular vein thrombus stemming from sphenoid sinusitis. She presented with lower-extremity weakness, and imaging revealed the thrombus. Subsequent intraoperative cultures identified *T. praeacuta*. Treatment encompassed intravenous antibiotics and anticoagulation. This case underscores the need to consider unusual pathogens in sinusitis-related thrombosis. To the authors’ knowledge, this report is the first documented case of *T. praeacuta *isolated in sinusitis leading to infectious thrombophlebitis, and it adds to the scarce literature on *T. praeacuta* infections, emphasizing a multidisciplinary approach for such complex cases.

## Introduction

*Tissierella praeacuta* is a Gram-negative anaerobic bacterium residing in the human gastrointestinal tract and in environmental sources [1]. Once thought to be a distinct organism, it is now classified as a member of the *Clostridium* subphylum [2]. Treatment options for infections by this bacterium remain unstandardized as it is a very rare cause of human infection. Cases in the literature include ovarian thrombophlebitis, pseudoarthrosis, osteomyelitis, brain abscess, sacral wounds, pyonephrosis, hepatic abscess, and gas gangrene of the eyelid [1,3-8].

Further complicating treatment for *Tissierella* is that identification can take six to seven days and most labs do not perform susceptibilities routinely [6,7]. Decisions on how to treat are usually based on data from previous case reports with options that include beta-lactam/lactamase inhibitor combos, fluoroquinolones, meropenem, rifampin, and metronidazole [1,3-8]. This presentation looks to add to the limited literature of *T. praeacuta* with the intent of further defining this bacterium as a potential cause of even more human infections and to aid in creating more standardized and evidence-based treatment regimens for practitioners to use if they are to ever encounter this bug.

## Case presentation

A 71-year-old female with a medical history significant for ischemic stroke, carotid artery stenosis, basilar artery stenosis, and previous neurological symptoms initially presented to the emergency room complaining of lower-extremity weakness that she noticed when trying to get out of bed. Since arriving at the ER, her symptoms had resolved. The patient had several similar episodes in the past over a six-month period; her symptoms consisted of bilateral lower-extremity weakness without sensory deficit, nausea, and lightheadedness. Previous neurological work-up to rule out acute strokes had been unrevealing and the patient was told that her previous episodes might have been caused by transient ischemic attacks (TIAs). On presentation, the patient was afebrile and hemodynamically stable; the physical exam was largely unremarkable, without neurologic deficits, although left cervical lymphadenopathy was noted. In the ER, her only laboratory abnormalities included a mildly elevated WBC of 11.4 K/uL and a creatinine of 1.0 (patient baseline 0.7-0.8). The patient was given clopidogrel and aspirin for possible CVA and seen by neurology to evaluate symptoms.

**Figure 1:**
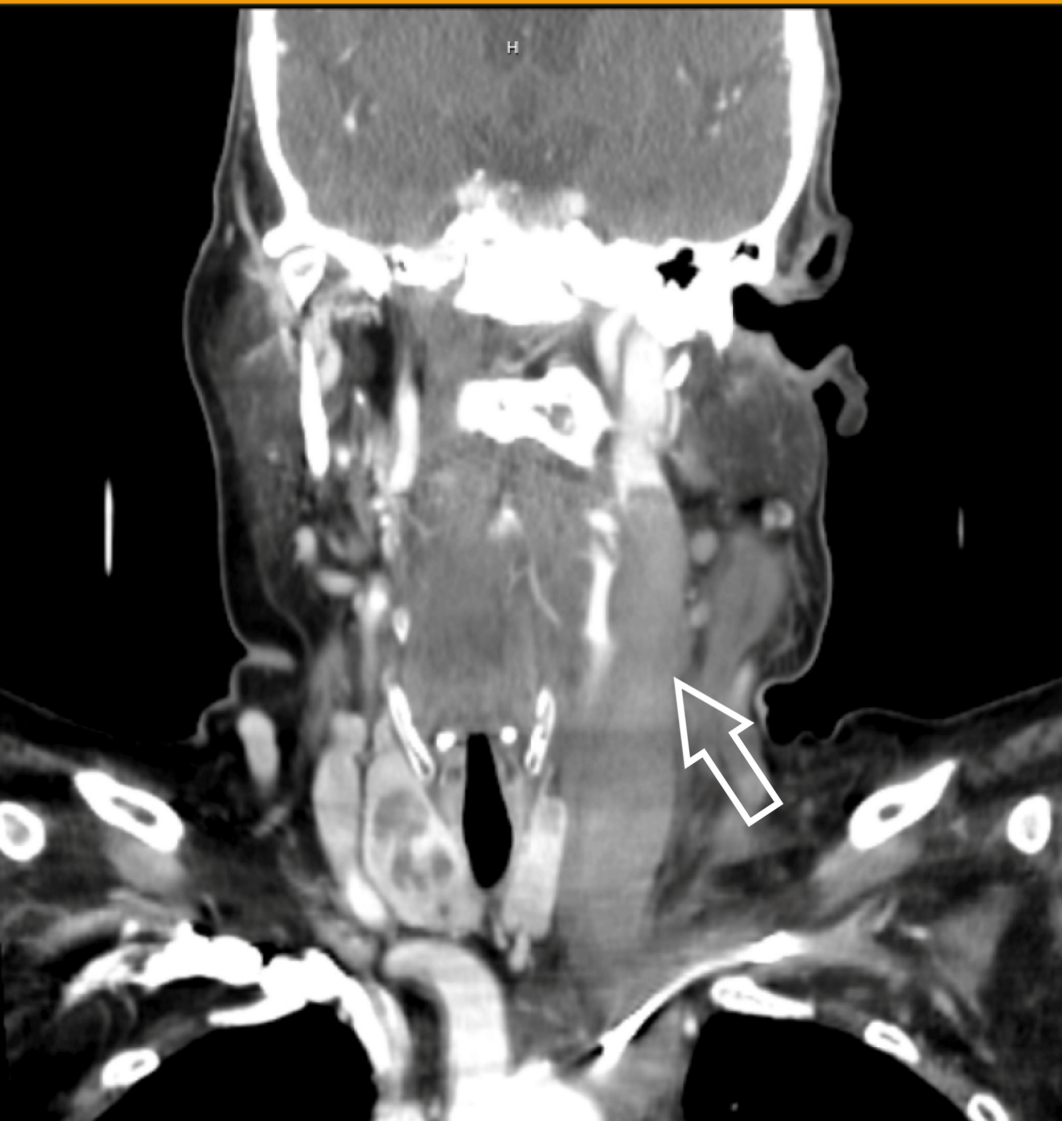
Thrombus of the internal jugular vein (white arrow) as seen on the coronal view of the computed tomography angiography (CTA) head and neck

A computed tomography angiography (CTA) of the head/neck showed asymmetric moderate stranding of the left internal jugular vein with concerns of thrombophlebitis/Lemierre's syndrome and mildly enlarged left-sided lymph nodes (Figure 1). These findings prompted a computed tomography (CT) neck (soft tissue), which revealed bilateral sphenoid/ethmoid sinusitis and thrombophlebitis of the left internal jugular vein extending from cervical vertebrae #2 to the brachiocephalic confluence. No other acute intracranial process was found. Otolaryngology was consulted and the diagnosis of Lemierre’s syndrome was confirmed. The patient was started on a heparin infusion and empiric antibiotic therapy with piperacillin/tazobactam and vancomycin. She then underwent urgent bilateral sphenoidotomy from which cultures were obtained. Postoperatively, the patient was brought to the intensive care unit (ICU) due to substantial bleeding from the nasopharynx, resulting in heparin reversal with protamine sulfate. Following cessation of bleeding and successful heparin re-initiation, the patient was moved to the floor.

Intraoperative cultures from the sinuses grew 1+ *Staphylococcus epidermidis*, 1+ *Streptococcus anginosus*, and 1+ *Prevotella bivia*; the predominant organism was 4+ *T. praeacuta*. With the exception of *T. praeacuta*, whose sensitivities have not resulted, the other organisms were all sensitive to vancomycin, and *S. anginosus* was sensitive to ampicillin as well. Fungal cultures from the sinuses were negative and blood cultures returned with no growth. Infectious disease was consulted; per recommendations, piperacillin-tazobactam was discontinued and therapy was narrowed to ampicillin/sulbactam, with continuation of vancomycin.

The patient was ultimately discharged to home 15 days after admission on a six-month course of apixaban and a 13-day course of amoxicillin-clavulanic acid. This will complete a total four-week course of antibiotics, and she was to follow up with the otolaryngologist for her chronic sinusitis. At present, the patient has improved back to her baseline functional status, and multiple outpatient physical therapy and primary care notes document a normal physical exam without neurologic deficits or complaints. 

## Discussion

Lemierre's syndrome is a rare and life-threatening condition with an estimated incidence of 3.6 cases per million annually [9] and a mortality rate of approximately 4-18% [10]. The syndrome typically follows upper airway or oropharyngeal infection, leading to septic thrombophlebitis of the internal jugular vein and potential systemic complications, such as lung abscesses [11]. The most common causative microorganism is *Fusobacterium necrophorum*, a gram-negative anaerobe, although other bacteria like *Enterobacteriaceae*, *Bacteroides*, and *Streptococci* may also be involved [12]. Risk factors for this syndrome include recent upper airway or oropharyngeal infections, underlying immunosuppression, and poor oral hygiene [13]. Mainstay treatment involves antibiotic therapy with anticoagulation being controversial as a non-extensive clot burden can resolve on its own with appropriate antibiotics and supportive care. For large or bilateral clots, or if the patient fails to show improvement after 72 hours of appropriate antibiotics, then anticoagulation is warranted [11]. This report documents a case of Lemierre's syndrome secondary to chronic sinusitis with the predominate bacterium being *T. praeacuta*.

There have only been two other documented cases in the literature of thrombophlebitis caused by *T. praeacuta*. One includes a 67-year-old female with pylephlebitis, septic portal vein thrombosis, who was treated with a heparin infusion IV ceftriaxone and metronidazole. She was ultimately discharged on four weeks of antibiotics and a three-month course of apixaban [14]. The other case included a 24-year-old female with septic ovarian thrombophlebitis following an uncomplicated vaginal delivery. She developed subsequent bacteremia and was treated with a heparin infusion, meropenem, and clindamycin while hospitalized. Upon discharge, she was given four weeks of metronidazole and amoxicillin/clavulanic acid along with three months of oral anticoagulation [7]. In both cases, repeat imaging was performed to ensure the resolution of thrombi, but our patient never followed up on this, although it was ordered, and repeat imaging was never performed. Our patient received similar treatment while hospitalized with initially broad coverage with piperacillin/tazobactam and vancomycin along with a heparin infusion. In light of reported sensitivities for* T. praeacuta*, prior case reports were used to guide antibiotic narrowing to ampicillin/sulbactam alone and then again to amoxicillin/clavulanic acid. Given her relatively long hospital stay, she finished half of her four weeks of total antibiotic therapy while inpatient, although this did not shorten her course of anticoagulation therapy, which was six months upon discharge. The reason for the protracted anticoagulation course was due to the size of the clot burden and location.

The underlying pathophysiology of Lemierre's syndrome is thought to involve the close proximity of the lymphatic vessels, which drain structures of the head, and lymph nodes to the internal jugular vein. This close anatomical relationship is thought to facilitate the spread of infections to the systemic circulation [13]. Given that *T. praeacuta* is found in the gastrointestinal tract and in the environment, we have to wonder how this patient's sinuses and subsequently a left IJ clot became infected with the organism. An interesting piece of history collected later in her hospital stay revealed that she had been hospitalized four months prior due to respiratory distress. At that time, there were concerns of a possible aspiration component secondary to dysphagia. It is plausible to believe that at some point she may have aspirated some gastrointestinal contents due to her dysphagia, which in turn could have introduced the bacteria to the sinuses. Although we may never know how her sinuses became infected with *T. praeacuta*, this case report serves to add to the limited literature on human infections caused by this bacterium.

## Conclusions

*T. praeacuta *is a rare cause of a variety of human infections; previous cases document successful treatment with beta-lactam/beta-lactamase inhibitor combinations, rifampin, meropenem, or metronidazole. Our patient was treated with a short course of piperacillin/tazobactam and then switched to ampicillin/sulbactam and vancomycin inpatient, followed by an outpatient course of amoxicillin-clavulanic acid. She also received a six-month course of anticoagulation therapy with apixaban. This course was different than previous case reports that used only piperacillin/tazobactam or meropenem while inpatient and then an outpatient regimen of levofloxacin or metronidazole. The regimen chosen for this patient was likely due to the polymicrobial infection found in intraoperative cultures. The large clot burden and location also explain the choice for a six-month course as opposed to a three-month course of anticoagulation therapy as seen in other cases of septic thrombi. Although it still remains unclear how she became initially infected with *T. praeacuta*, this case contributes to the limited existing literature regarding human infections caused by the bacterium. A literature review is warranted as there remain no standardized or guideline-directed treatment plans for these infections.
